# Supplementary material for: Role of Combined Na2HPO4 and ZnCl2 in the Unprecedented Catalysis of the Sequential Pretreatment of Sustainable Agricultural and Agro-Industrial Wastes in Boosting Bioethanol Production
Source: Int J Mol Sci. 2022 Feb 4;23(3):1777. doi: 10.3390/ijms23031777 (PMC8836262; doi:10.3390/ijms23031777)
Supplement: Supplementary file 1 [file ijms-23-01777-s001.zip › ijms-1528539-supplementary.pdf]

Supplementary information

Role of combined Na_2HPO_4 and $ZnCl_2$ in the unprecedented catalysis of the sequential pretreatment of sustainable agricultural and agro-industrial wastes in boosting bioethanol production

Shaimaa Elyamny [1], Ali Hamdy [2], Rehab Ali [3,*], and Hesham Hamad [3,*]

[1] Electronic Materials Research Department, Advanced Technology and New Materials Research Institute (ATNMRI), City of Scientific Research and Technological Applications (SRTA-City), Alexandria 21934, Egypt; selyamny@srtacity.sci.eg
[2] Environmental Biotechnology Department, Genetic Engineering and Biotechnology Research Institute (GEBRI), City of Scientific Research and Technological Applications (SRTA-City), Alexandria 21934, Egypt; ali.hamdy343@yahoo.com
[3] Fabrication Technology Research Department, Advanced Technology and New Materials Research Institute (ATNMRI), City of Scientific Research and Technological Applications (SRTA-City), Alexandria 21934, Egypt;
* Correspondence: rehabmohamedali1983@gmail.com, rali@srtacity.sci.eg (R.A); heshamaterials@hotmail.com, hhamad@srtacity.sci.eg (H.H)

S1. Physico-chemical characterization methods

The TP, CS, and their pretreated samples were characterized and analyzed using different techniques. The samples composition from cellulose, hemicellulose and lignin was estimated using the modified Van Soest method [56]. Fourier transform infrared spectroscopy (FTIR, Shimadzu-8400S, Japan) was used to identify the chemical bonds onto the samples' surface, functional groups, and their chemical structure. The FTIR analysis was performed by mixing 2 mg of each sample with 100 mg of potassium bromide KBr (Sigma-Aldrich, Darmstadt, Germany) grinding to uniform particle size, and pressing as a pellet using a hydraulic press to be analyzed [39]. Structural properties and crystalline of the C and T and their pretreated samples were examined using X-ray diffraction (XRD, Shimadzu 7000, Japan). The patterns were recorded at CuK_α radiation wavelength (λ= 1.5418 Å), generated at voltage 30 kV and a filament emission of 30 mA with a step-scan mode; scanning rate of 5° min^{-1}, 2θ values range: 5 - 80°, step-time: 0.5 s, step-width: 0.1°. The surface morphology, homogeneity, and surface features were investigated using a scanning electron microscope (SEM JEOL, JSM-6360 LA, Japan) at room temperature and accelerating voltage 20 kV. The thermal stability of the samples was performed using (SDT Q600 V20.9 Build 20 Instrument). The investigations were conducted in a nitrogen atmosphere at a rated flow of 20 ml/min, from room temperature to 800°C at a heating rate of 10°C/min.

S2. Determination of surface functional groups

Boehm's acid-base titration experiments were conducted to determine the surface chemistry of the sample [57]. Briefly, a mass of 0.1 g sample was added to each of four volumetric flasks (100 mL) containing 50 ml (0.1 M) of Na_2CO_3, $NaHCO_3$, NaOH, and HCl solutions, respectively. The flasks were sealed and mechanically stirred for 48 h under room temperature conditions. After 48 h, the solutions were filtered through 0.45 μm nylon filters. Then, 10 mL of filtrate from each flask were titrated with 0.1 M HCl (for excess base) and 0.1 M NaOH (for excess acid).

S3. Glucose yield estimation

The glucose yield produced by the aid of the enzymes was estimated by centrifugation of 1 ml hydrolysate to separate supernatant, then 10 ul of supernatant was added to 1 ml of glucose buffer. The mixture was incubated for 15 min at room temperature and the absorbance was determined at λ_{max} 546 nm using (Spectrophotometer 7230 G, Shanghai, China).

S4. Yeast isolation

Yeast isolates were collected from different sources; rotten banana and orange. About 0.1 gm of each sample was serially diluted in a reaction tube containing 1 ml of sterile saline solution. About 500 µl of the serial dilutions were spread on a petri dish containing a yeast extract peptone agar (YEPA) medium with a composition of 20 gm peptone, 20 gm glucose, 10 gm yeast extract, and 20 gm agar. Then the cultures were incubated at 30˚C for 48 hours. All yeasts colonies were then purified and screened by using a specific medium to determine the purity of the isolated yeast colonies.
